# Validation of an Index for Functionally Important Respiratory Symptoms among Adults in the Nationally Representative Population Assessment of Tobacco and Health Study, 2014–2016

**DOI:** 10.3390/ijerph18189688

**Published:** 2021-09-14

**Authors:** Michael J. Halenar, James D. Sargent, Kathryn C. Edwards, Steven Woloshin, Lisa Schwartz, Jennifer Emond, Susanne Tanski, John P. Pierce, Kristie A. Taylor, Kristin Lauten, Maciej L. Goniewicz, Raymond Niaura, Gabriella Anic, Yanling Chen, Priscilla Callahan-Lyon, Lisa D. Gardner, Theresa Thekkudan, Nicolette Borek, Heather L. Kimmel, K. Michael Cummings, Andrew Hyland, Mary F. Brunette

**Affiliations:** 1 Westat, Rockville, MD 20850, USA; KatyEdwards@westat.com (K.C.E.); KristieTaylor@westat.com (K.A.T.); KristinLauten@westat.com (K.L.); 2 Geisel School of Medicine at Dartmouth, The C. Everett Koop Institute at Dartmouth, Hanover, NH 03755, USA; James.D.Sargent@dartmouth.edu (J.D.S.); Jennifer.A.Emond@dartmouth.edu (J.E.); Susanne.E.Tanski@dartmouth.edu (S.T.); Mary.F.Brunette@hitchcock.org (M.F.B.); 3 Dartmouth Institute for Health Policy and Clinical Practice, The C. Everett Koop Institute at Dartmouth, The Lisa Schwartz Foundation, Lebanon, NH 03766, USA; Steven.Woloshin@dartmouth.edu (S.W.); Lisa.Schwartz@Dartmouth.edu (L.S.); 4 Moores Cancer Center, University of California at San Diego, La Jolla, CA 92037, USA; jppierce@health.ucsd.edu; 5 Department of Health Behavior, Roswell Park Comprehensive Cancer Center, Buffalo, NY 14203, USA; Maciej.Goniewicz@roswellpark.org (M.L.G.); andrew.hyland@roswellpark.org (A.H.); 6 School of Global Public Health, New York University, New York, NY 10012, USA; rn54@nyu.edu; 7 Office of Science, Center for Tobacco Products, Food and Drug Administration, Silver Spring, MD 20993, USA; Gabriella.Anic@fda.hhs.gov (G.A.); Yanling.Chen@fda.hhs.gov (Y.C.);Priscilla.Callahan-Lyon@fda.hhs.gov (P.C.-L.); Lisa.Wasson@fda.hhs.gov (L.D.G.); Theresa.Thekkudan@fda.hhs.gov (T.T.); Nicolette.Borek@fda.hhs.gov (N.B.); 8 National Institute on Drug Abuse, National Institutes of Health, Bethesda, MD 20852, USA; Heather.Kimmel@nih.gov; 9 Department of Psychiatry & Behavioral Science, Medical University of South Carolina, Mt. Pleasant, SC 29464, USA; cummingk@musc.edu

**Keywords:** tobacco use, respiratory health, wheeze, functional outcomes, patient-reported outcomes, PATH Study

## Abstract

The purpose of this study is to validate the seven-item wheezing module from the International Study of Asthma and Allergies in Children (ISAAC) in the nationally representative Population Assessment of Tobacco and Health Study. Adult participants with complete Wave 2–3 data were selected, including those with asthma but excluding those with COPD and other respiratory diseases (*n* = 16,295). We created a nine-point respiratory symptom index from the ISAAC questions, assessed the reliability of the index, and examined associations with self-reported asthma diagnosis. Threshold values were assessed for association with functional outcomes. The weighted prevalence for one or more respiratory symptom was 18.0% (SE = 0.5) for adults without asthma, 70.1% (SE = 1.3) for those with lifetime asthma, 75.7% (SE = 3.7) for adults with past-year asthma not on medications, and 92.6% (SE = 1.6) for those on medications. Cronbach’s alpha for the respiratory symptom index was 0.86. Index scores of ≥2 or ≥3 yielded functionally important respiratory symptom prevalence of 7–10%, adequate sensitivity and specificity for identifying asthma, and consistent independent associations with all functional outcomes and tobacco use variables. Respiratory symptom index scores of ≥2 or ≥3 are indicative of functionally important respiratory symptoms and could be used to assess the relationship between tobacco use and respiratory health.

## 1. Introduction

The focus of this report is respiratory symptoms (wheezing and dry cough) indicative of airway obstruction. Wheezing is an important respiratory symptom suggesting the existence of asthma and/or chronic bronchitis [1]. Its association with cigarette smoke exposure, especially among young people [2], makes this an important endpoint in any study of tobacco use and disease. There are several validated questionnaires for assessing wheezing in children [3,4] or adults [5] but not typically for both populations.

The Population Assessment of Tobacco and Health (PATH) Study is an ongoing, nationally representative, longitudinal cohort study that obtains detailed information on tobacco use in the U.S. population. The PATH Study aims to assess the relationship between tobacco product use and health outcomes. Respondents are asked questions about their health, including self-reports of chronic diseases such as diabetes, heart attacks, stroke, cancer, respiratory disease, disease symptoms, and functional outcomes [6]. 

The PATH Study adult interview includes a core wheezing assessment from the International Study of Asthma and Allergies in Children (ISAAC) that was validated and used widely in children [7,8,9], and modified for use with adults [10,11]. The goal of this report is to create a validated respiratory symptom index from the included ISAAC questions in a sample of adult asthmatics, as well as in a large representative sample of the adult general population (including asthmatics).

We also sought to assist researchers in assigning a respiratory symptoms index cut-off value that would serve as a binary indicator of functionally important respiratory symptoms, which was validated against self-reported asthma diagnosis and functional outcomes. By exploring how various respiratory symptom index cut-off values relate to asthma diagnoses and functional outcomes such as fatigue, researchers can choose a dichotomous outcome indicative of functional impairment. The respiratory symptoms index and potential cut-off values should provide estimates of impairment or disease that align with existing literature. Since approximately 10–15% of the adult population has significant obstructive impairment [12] and about 8% of the adult population has been diagnosed with asthma [13,14], we will evaluate if our index yields comparable estimates of impairment. 

Finally, the association between current cigarette smoking, cigarette smoking history, and second-hand smoke exposure with the respiratory symptom index was examined using PATH Study data. Our hope is that researchers will be able to confidently use this index to study the effects of tobacco use on respiratory health.

## 2. Materials and Methods

### 2.1. Study Population

Recruitment for Wave 1 (W1; 2013–2014) of the PATH Study employed stratified address-based, area-probability sampling with oversampling of adult tobacco users, young adults (18 to 24 years), and African-Americans. An in-person screener selected adults from households at W1, and audio computer-assisted self-interviews collected data for tobacco-use and health outcomes. Respiratory symptoms were assessed in Waves 2 (W2; 2014–2015) and 3 (W3; 2015–2016), including 28,362 and 28,148 adult participants, respectively (weighted response rates of 83.2% and 78.4%, respectively). Our analyses utilized the adult W2 and W3 Restricted Use Files (https://doi.org/10.3886/ICPSR36231.v21 (accessed on 17 February 2021)). 

**Figure 1:**
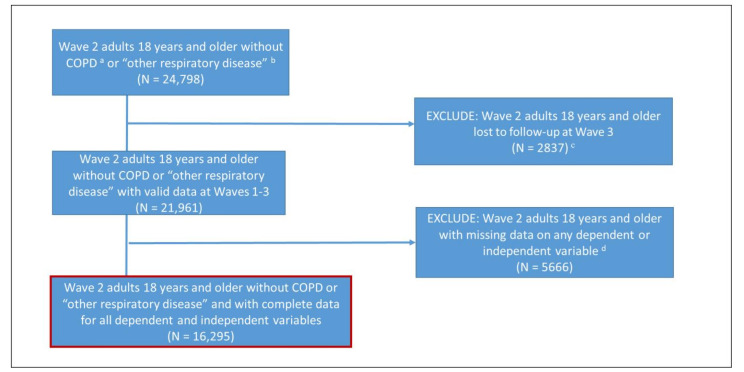
Main analytic sample determination. This figure illustrates the sample determination from the Population Assessment of Tobacco and Health Study for the main analyses that this validation will be used as described in the Materials and Methods of the manuscript. Some functional outcome analyses have additional variables that reduce the sample for those analyses further due to missingness. ^a^ Chronic obstructive pulmonary disease. ^b^ Other non-asthma respiratory diseases. ^c^ Weights adjust for non-response. ^d^ Dependent variables include Respiratory Symptom Index at Waves 2 and 3. Independent variables include tobacco product use at Waves 2 and 3 along with Wave 2 determinations of asthma, cigarette pack years, age, sex, ethnicity/race, education, income, congestive heart failure, diabetes, cancer, heart attack, body mass index, marijuana use, past week second-hand smoke exposure, regular use of Beta Blockers, Angiotensin Receptor Blockers, or Ace Inhibitors, and Wave 1 determination of urbanicity.

The validation sample included in these analyses corresponds to the sample selected for a separate manuscript, which utilized a complete case analysis to evaluate the association between tobacco use and changes in respiratory symptoms in the general population of adults, including adults with asthma [15], which will be referred to as the general population moving forward (see Figure 1). At W2, there were 24,798 adults without self-reported health professional diagnosis of chronic obstructive pulmonary disease (COPD) or other non-asthma respiratory diseases by Wave 3. Adults with COPD or other non-asthma respiratory disease were excluded because those are non-reversible (or unknown) lung diseases and might skew the ability to validate the respiratory symptom scale for the general population. The exclusion criteria outlined in Figure 1 include (1) participants lost to follow-up at W3 (*n* = 2837, 11%) and (2) those with missing data on any analytic variables (*n* = 5666, 23%) used in the aforementioned manuscript and described more in the Figure 1 footnotes, for a final analytic sample of 16,295. Validation results in this report did not change when using the full sample (i.e., not excluding those with missing data on any analytic variables; *n* = 21,961). All analytic variables in this report come from W2 (2014–2015) except for when evaluating test–retest reliability, as described below. PATH Study design and methods [6,16,17], interviewing procedures, questionnaires, sampling, weighting, and response rates are in the *PATH Study Restricted Use Files User Guide* [18]. The study was conducted by Westat and approved by the Westat Institutional Review Board. All respondents provided informed consent.

### 2.2. Measures for Respiratory Symptoms in the PATH Study

The core wheezing module from the ISAAC [7] is used to assess wheezing and nighttime cough in children and adults in the PATH Study. The seven ISAAC questions (Table 1) have been validated in children and widely implemented across many countries [8,9].

### 2.3. Item-Level Validation against Similar NHANES Items

Five of the ISAAC items are similar to those used to assess respiratory symptoms in the National Health and Nutrition Examination Survey (NHANES). Weighted prevalence estimates were calculated for ISAAC items in W2 (2013–2014) of the PATH Study and compared with similar items from the 2011–12 NHANES. 

### 2.4. Creating a Respiratory Symptoms Index

Since combining similar items in an index increases its reliability [19], responses to the seven ISAAC core wheezing module questions were used to create a respiratory symptoms index. Three wheezing variables (ever wheezing, past 12-month (P12M) wheezing, and P12M wheezing attack frequency) were combined to create one variable (0 = never wheezing; 1 = ever wheezing, but no P12M wheezing OR P12M wheezing but no wheezing attacks; 2 = P12M wheezing and 1–3 attacks; 3 = P12M wheezing and 4–12 attacks; 4 = P12M wheezing and more than 12 attacks). This new variable was summed with remaining four questions (three of which are yes = 1/no = 0, and one of which has three answer choices scored as 0, 1, or 2) to create the index ranging from 0 to 9, where 0 represented no respiratory symptoms, and 9 represents the highest level of symptoms (i.e., a “yes” to all dichotomous outcomes and the highest level for questions with multiple responses).

Internal consistency of the index was examined using Cronbach’s alpha. Test–retest reliability was assessed by pairwise correlation between respiratory symptoms index scores at W2 with scores approximately one year later at W3. 

### 2.5. Validating the Respiratory Symptoms Index against Asthma 

Then, we examined the association between index scores and self-reported health professional-diagnosed asthma. Since wheezing is a key feature of asthma, using the index to predict asthma diagnosis is useful to support construct validity and the possibility for utilizing the index within the general population as well. Lifetime asthma diagnosis was indicated by a response of asthma to the following question, “Has a doctor, nurse or other health professional ever told you that you had any of the following lung or respiratory conditions?” Individuals with lifetime asthma were asked if they had asthma in the past 12 months and whether they had taken any medication for asthma in the past 12 months. Those with P12M asthma were divided into two groups based on whether they had taken any asthma treatment medications. The weighted proportion with each type of asthma was examined as a function of their respiratory symptom index score. 

### 2.6. Determining Cut-Off Values for Functionally Important Respiratory Symptoms among Those with Asthma

Weighted logistic regression and unweighted receiver operator characteristic (ROC) curves were used to examine the association between the respiratory symptoms index at various cut-off levels with the three asthma outcomes (ever, P12M without medications, P12M with medications), reporting prevalence, sensitivity, the false positive rate, and the unadjusted association with asthma for cut-off values of ≥1, ≥2, ≥3, and ≥4. ROC curves allow researchers interested in defining thresholds for disease to understand the tradeoff between sensitivity (i.e., the ability to correctly include all with the disease outcome of interest) and the false positive rate (i.e., those without the disease of interest who are included in the test positive subpopulation) [20]. 

### 2.7. Association between Respiratory Symptoms and Functional Outcomes in the General Population

PATH Study respondents were also questioned about functional outcomes (e.g., physical limitations, fatigue, and general health) with items from the Patient-Reported Outcomes Measurement Information System (PROMIS) physical health question bank [21]. The questions captured activity limitations (e.g., health limits walking 3 blocks), fatigue in the past 7 days, and general physical health, and they have high reliability [22]. 

Weighted logistic regression or multinomial logistic regression was used to assess the relationship between respiratory symptom scores (as a continuous outcome and at cut-off values ≥1, ≥2, ≥3, and ≥4) and functional outcomes. First, we inspected the association between higher scores on the respiratory symptoms index and the proportion with functional limitations, looking for nonlinearity. Next, we tested the independent relationship of both the continuous index and dichotomous measures of functionally important respiratory symptoms with functional outcomes using weighted multivariable logistic or multinomial regression, adjusting for demographics and other diseases (diabetes, cancer, congestive heart failure, and heart attack) that could confound the association. 

Finally, weighted multivariable logistic regression was used to test the independent associations between both the continuous respiratory symptom index and functionally important symptoms at the four cut-off levels and three tobacco outcomes that have been previously associated with wheezing: current cigarette smoking (never, former, past 30-day nondaily, or past 30-day daily), pack-years of cigarette smoking (never smokers were assigned a value of 0; entered as a continuous measure with each 1-point increase indicative of an additional 5 pack-years of smoking), and second-hand smoke exposure (with each 1-point increase indicative of an additional hour per week of exposure). 

### 2.8. Analytical Approach

All main analyses were weighted using the W3 longitudinal (all-waves) full-sample and replicate weights to adjust for the complex sample design and loss to follow up. Variances were estimated using the BRR method [23] with Fay’s adjustment set to 0.3 to increase estimate stability [24]. Pack-years of cigarette smoking and second-hand smoke exposure were Winsorized at the 95th and 99th percentiles respectively to address outliers [25]. All analyses used Stata survey data procedures, version 16.1 (StataCorp LLC, College Station, TX, USA) [26].

## 3. Results

### 3.1. Descriptive Statistics, Internal Consistency, and Test–Retest Reliability

The weighted prevalence for one or more index respiratory symptoms was 18.0% (standard error (SE) = 0.5) for adults without asthma, 70.1% (SE = 1.3) for those with lifetime asthma, 75.7% (SE = 3.7) for adults with asthma in the past year but not on medications, and 92.6% (SE = 1.6) for past-year asthmatics on medications. For the same four categories, the weighted mean (SE) for the respiratory symptoms index was 0.3 (0.0), 2.0 (0.1), 2.2 (0.1), and 3.6 (0.1) respectively. The respiratory symptom index had acceptable internal consistency (Cronbach’s alpha = 0.86) and one-year test–retest reliability (r = 0.72 for pairwise correlation between W2 and W3 results).

### 3.2. Item-Level Validation against Similar NHANES Questions

Table 1 reports weighted prevalence for respiratory symptoms based on W2 of the PATH Study compared to NHANES 2011–2012 among adults 18+ years old. The table shows similar-to-higher prevalence of past-year wheezing based on PATH Study W2 compared to NHANES. For example, the prevalence of disturbed sleep due to wheezing one or more nights per week was ≈3% in both studies, whereas wheezing in the past 12 months was higher in the PATH Study than NHANES (16.8% vs. 13.1%). The prevalence of dry cough at night was much higher in the PATH Study, which was most likely due to differences in time frame (at least 14 days of persistent cough for the NHANES question compared to no timeframe for the PATH Study question). 

### 3.3. Association of Respiratory Symptoms Index with Self-Reported Asthma

**Figure 2:**
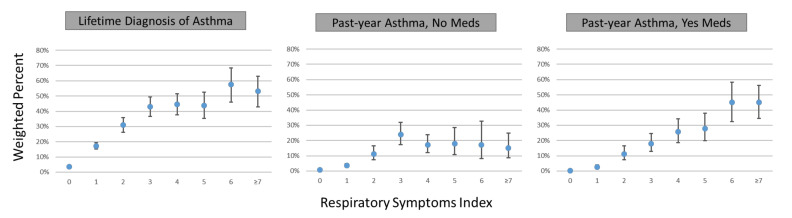
Weighted relationship between respiratory symptoms index score and self-reported asthma, Wave 2 (2014–2015) of the Population Assessment of Tobacco and Health (PATH) Study. PATH Study asthma outcomes illustrated in the figure: Lifetime Asthma (Unweighted *n* = 1829/16,295). Past-Year Asthma, No Meds (Unweighted *n* = 436/14,902). Past-Year Asthma, Yes Meds (Unweighted *n* = 371/14,837.

Figure 2 illustrates the association between higher scores on the respiratory symptoms index and self-reports of asthma diagnosis (respiratory symptom scores of 7–9 were collapsed because of small numbers in those categories). Less than 4% of persons with no symptoms had any asthma diagnosis. For lifetime and past-year asthma without medications, the association was nonlinear, increasing up to an index score of 3 with less consistent change thereafter (suggesting a cut-off threshold of ≥3); in contrast, the association was roughly linear for past year asthma with medications, increasing from 0.2% (95% confidence interval (CI) 0.1, 0.3) for those with no symptoms to >45% for those with scores of 6 or more.

### 3.4. Association of Various Respiratory Symptom Score Cut-Off Values with Self-Reported Asthma

Results from the ROC analysis (Table 2) indicated that the respiratory symptoms index was strongly associated with asthma (area under the ROC curve = 0.75, 0.80, and 0.90 for lifetime asthma, past-year asthma on no medications, and past-year asthma on medications, respectively). Symptom prevalence was upwards of 20% for a threshold of ≥1, 10.7% for ≥2, 7.2% for ≥3, and less than 5% for a threshold of ≥4. Sensitivity was best for more severe asthma; for example, for a threshold of ≥3, sensitivity was 35.2%, 45.8%, and 67.7% respectively for lifetime asthma, past-year asthma on no medications, and past-year asthma on medications. In classifying past-year asthma on medications, thresholds of ≥1 through ≥4 were associated with sensitivities of 91.9%, 80.1%, 67.7%, and 54.2%, respectively. The false positive rates were approximately 22% for a threshold of ≥1 and 10% and 7% for thresholds of ≥2 and ≥3, respectively. Unadjusted odds ratios (OR) for the association with asthma indicated strong associations that increased as expected with recency and severity of asthma diagnosis (e.g., for a threshold of ≥3, the ORs were 12.37 (95% CI 10.55, 14.49), 17.85 (95% CI 13.13, 24.28), and 43.62 (95% CI 31.24, 60.90) for lifetime asthma, past-year asthma on no medications, and past-year asthma on medications, respectively).

### 3.5. Association of Respiratory Symptom Score Cut-Off Values with Functional Outcomes in the General Population

The functional outcome questions and their weighted prevalence in the adult population are shown in Table 3. Whereas 4.2% experienced health limits for walking up 10 steps, 9.4% experienced limits for walking a mile. Only 23.8% of adults experienced no fatigue, but only 4.0% and 0.1% respectively experienced severe or very severe fatigue. Similarly, only 17.4% were in excellent health, but only 9.3% and 1.1% respectively experienced fair or poor health. 

**Figure 3:**
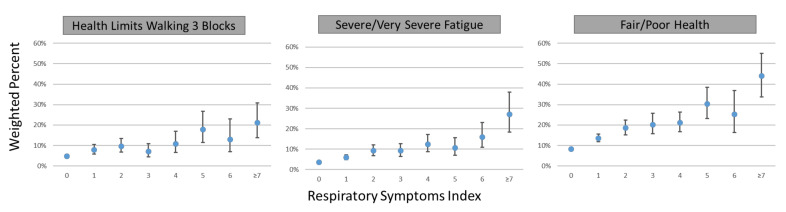
Weighted relationship between respiratory symptoms index score and functional outcomes, Wave 2 (2014–2015) of the Population Assessment of Tobacco and Health (PATH) Study. PATH Study functional outcomes illustrated in the figure: Does your health limit you in any of the following activities? Walking three blocks. (Unweighted *n* = 16,263). In the past 7 days, how would you rate your fatigue on average? By fatigue, we mean feeling unrested or overly tired during the day, no matter how many hours of sleep you have had. (Unweighted *n* = 16,284). In general, how would you rate your physical health? (Unweighted *n* = 16,291).

The associations between higher scores on the respiratory symptoms index and three functional outcomes—health limits walking three blocks, severe/very severe fatigue, and fair/poor health—are illustrated in Figure 3. The percentage of people with health limits walking three blocks increased from 4.7% (95% CI 4.1, 5.5) for adults with no respiratory symptoms to 21.1% (95% CI 13.9, 30.9) for those with scores of ≥7. The percentage with severe to very severe fatigue increased from 3.6% (95% CI 3.2, 4.1) for those with no symptoms to 27.0% (95% CI 18.3, 37.9) with scores of ≥7, and fair to poor health increased from 8.2% (95% CI 7.5, 9.0) to 44.1% (95% CI 33.7, 55.0). Finally, there was no visual evidence of a respiratory symptom threshold for functional impairment; instead, increases in functional impairment were seen across the spectrum of the respiratory symptoms index.

Table 4 shows the independent association between the continuous index or various respiratory symptom index cut-off levels and functional outcomes. Regardless of cut-off level chosen, persons with scores above the cut-off level had a higher risk of all functional outcomes. For the continuous index, all relationships were significant, but with lower estimates (ORs or risk ratios) than when using any of the cut-points. For the physical outcomes such as walking three blocks, the adjusted ORs increased from 1.25 (95% CI 1.18, 1.33) as a continuous index to 1.77 (95% CI 1.41, 2.22) among those with a score of ≥1 and to 2.89 (95% CI 2.13, 3.93) among those with a score of ≥4. Estimates tended to be higher with higher cut-off levels. For example, the OR for difficulty in walking a mile increased from 2.00 (95% CI 1.65, 2.43) to 3.13 (95% CI 2.39, 4.10) as the cut-off value increased from ≥1 to ≥4. Higher ORs were also observed for more difficult function tasks at a given cut-off value; for example, at a cut-off value of ≥3, the ORs for difficulty walking up 10 steps was 2.00 (95% CI 1.45, 2.76) compared to 2.71 (95% CI 2.14, 3.43) for difficulty walking a mile.

Similar patterns were found in the multinomial regressions that examined the independent associations between various cut-off levels and more fatigue and poorer physical health. In no case was the independent association between respiratory symptoms and functional outcomes not statistically significant, and in many cases, the relative risks were above 2.00. For very severe fatigue vs. none, the relative risks were 1.74 (95% CI 1.54, 1.96), 3.82 (95% CI 2.37, 6.17), 6.31 (95% CI 3.94, 10.11), 7.69 (95% CI 4.73, 12.49), and 10.00 (95% CI 5.52, 18.11) for continuous and then cut-off levels of ≥1, ≥2, ≥3, and ≥4 respectively, and for a cut-off of ≥3, the relative risks increased from 1.83 (95% CI 1.41, 2.37) to 7.69 (95% CI 4.73, 12.49) when comparing mild, moderate, severe, and very severe fatigue against none. For poor physical health vs. excellent physical health, the relative risks were 1.91 (95% CI 1.73, 2.11), 6.70 (95% CI 4.46, 10.07), 8.64 (95% CI 5.78, 12.93), 9.02 (95% CI 5.88, 13.83), and 11.66 (95% CI 7.21, 18.83) for continuous and then cut-off levels of ≥1, ≥2, ≥3, and ≥4 respectively.

### 3.6. Association of Various Respiratory Symptoms Score Cut-Off Values with Cigarette Smoke Exposure in the General Population

Table 5 shows the association between various indicators of cigarette smoking exposure and respiratory symptom index as continuous and at the four cut-off levels. Important associations are found consistently for all measures of former and current cigarette use as well as pack-years of cigarette smoking and second-hand smoke exposure. The associations changed little as a function of which cut-off level was chosen, with the exception of current daily smoking, for which the relative risk is 2.29 (95% CI 2.08, 2.53) at a threshold of ≥1 and almost double that (4.31 (95% CI 3.36, 5.52)) at a threshold of ≥4.

## 4. Discussion

In this large nationally representative sample of adults without COPD, respiratory symptoms (wheezing and dry cough) indicative of airway obstruction were strongly associated with an asthma diagnosis and also related to functional impairments in the general population—including difficulty with physical tasks such as walking up stairs, increased levels of fatigue, and poorer perceptions of physical health. The association between higher scores on the respiratory symptoms index and functional impairment was linear, with those having more symptoms being more likely to be impaired, and the association was also independent of sociodemographics and other conditions predictive of functional impairment. We conclude that this index is a reliable and compact measure of respiratory symptoms in epidemiologic samples of adults without COPD.

With regard to guiding researchers toward a cut-off level indicative of functional impairment, relative risks for the association ranged from 2 to 11, depending on the functional outcome and the cut-off value chosen. Our results did not suggest a specific cut-off value for this index indicative of functionally important respiratory symptoms; indeed, all cut-off values from ≥1 to ≥4 were associated with both asthma and functional impairment. However, there are considerations that might narrow the choice for a cut-off threshold. Specifically, for the association between the respiratory symptoms index and lifetime and current asthma, a cut-off of ≥3 was suggested by the nonlinear association such that symptom scores began to level off after a score of ≥3.

Considering the prevalence of functionally important respiratory symptoms, a cut-off value of ≥1 seemed too inclusive, because it returned a prevalence of over 20%, whereas prevalence studies find that significant obstructive respiratory impairment is between 10 and 15% of the adult population [12]. One study using NHANES, where spirometry was used to diagnose obstructive lung disease, found that severe or very severe airway obstruction (FEV1% predicted <50%) was present in 3–5% of adults without asthma/COPD and 14–23% of those with the diagnosis of asthma/COPD [27]. Prevalence considerations suggest a cut-off level of ≥2 or ≥3, which result in prevalence consistent with the prevalence of asthma, which is about 8% of the adult population [13,14]. Another consideration is that higher cut-off levels identify more severe symptoms but increase the chances of missing significant disease, for example current asthma diagnosis with use of medications. ROC analysis confirmed that a cut-off of ≥2 or ≥3 offers the best tradeoff between sensitivity and specificity for the diagnosis of asthma. This study demonstrates that all cut-off levels show robust, independent associations with functional outcomes.

Additional analyses showed a significant relationship between the respiratory symptoms index and cigarette smoking exposure, pack-years of cigarette smoking, and second-hand smoke exposure. This index could provide researchers with a validated respiratory outcome in cross-sectional and longitudinal studies of tobacco use when more clinical measures are not available. Results showed that researchers could use the index as a continuous measure or use any of the four cut-off values presented.

Strengths of this study are the inclusion of a large, nationally representative sample of adults for the validation process, along with an extensive analysis that includes examination of index reliability, its association with self-reported asthma diagnosis, and its association with functional outcomes. As a large population study, there are weaknesses in that the study relies solely on self-report data. In addition, there was no medical record confirmation of the asthma diagnosis, and we were unable to make associations between self-reported wheezing symptoms and more objective measures such as spirometry. These limitations were addressed by comparing PATH Study data to that of NHANES and using questions already validated for use in capturing respiratory illness in other populations.

## 5. Conclusions

This respiratory symptoms index, based on questions that have been extensively developed and tested in children, serves as an adequate population-level instrument for assessing functionally important respiratory symptoms, indicative of airway obstruction, in this large nationally representative sample of adults without COPD. Additionally, possible thresholds for determining “functionally important respiratory symptoms” could be used to assess the relationship between exposures, such as tobacco product use, and respiratory illness. Analyses confirmed that a cut-off of ≥2 or ≥3 offers the best tradeoff between sensitivity and specificity for the diagnosis of asthma, although researchers may wish to use this data to create a different cut-off value depending on their research questions.

## Figures and Tables

**Table 1:** Respiratory symptom questions in the Wave 2 (2014–2015) PATH Study interview, along with similar questions in the NHANES 2011–2012 survey.

Wave 2 PATH Study 2014–2015					NHANES 2011–2012				
Question ^a^	Response Options	PATH Study Variable	Prevalence ^b^ (SE) 18+ Years		Question	Response Options	NHANES Variable	Prevalence ^c^ (SE) 18+ Years	
Have you ever had wheezing or whistling in the chest at any time in the past?	Yes, No	R02_ AX0046	Yes	26.5% (0.4)	None equivalent				
Have you had wheezing or whistling in the chest in the past 12 months?	Yes, No	R02_ AX0047	Yes	16.8% (0.3)	In the past 12 months, have you had wheezing or whistling in your chest?	Yes, No	RDQ070	Yes	13.1% (0.9)
How many attacks of wheezing have you had in the past 12 months?	None, 1–3, 4–12, more than 12	R02_ AX0048	None ^d^ 1–3 4–12 >12	84.6% (0.3) 9.7% (0.3) 3.2% (0.2) 2.5% (0.1)	In the past 12 months, how many attacks of wheezing or whistling have you had?	Count, if 12 or more episodes enter 12	RDQ080	None ^e^ 1–3 4–12+	87.3% (0.9) 7.3% (0.7) 5.4% (0.6)
In the past 12 months, how often, on average has your sleep been disturbed due to wheezing?	None, less than one night/week, one or more nights/week	R02_ AX0049	None ^d^ <1/w ≥1/w	93.4% (0.2) 4.1% (0.2) 2.5% (0.1)	In the past 12 months, how often, on average has your sleep been disturbed because of wheezing?	None, less than 1 night/week, one or more nights per week	RDQ090	None ^e^ <1/w ≥1/w	93.9 (0.6) 2.9% (0.4) 3.2% (0.4)
In the past 12 months, has wheezing ever been severe enough to limit your speech to only one or two words between breaths?	Yes, No	R02_ AX0050	Yes	2.3% (0.1)	None equivalent				
In the past 12 months, has your chest sounded wheezy during or after exercise?	Yes, No	R02_ AX0052	Yes	11.5% (0.3)	In the past 12 months, has your chest sounded wheezy during or after exercise or physical activity?	Yes, No	RDQ100	Yes ^e^	6.7% (0.7)
In the past 12 months, have you had a dry cough at night, apart from a cough associated with a cold or chest infection?	Yes, No	R02_ AX0053	Yes	16.5% (0.3)	In the past 12 months, have you had a dry cough at night not counting a cough associated with a cold or chest infection for at least 14 days?	Yes, No	RDQ140	Yes	5.0% (0.4)

Abbreviations: PATH = Population Assessment of Tobacco and Health; NHANES = National Health and Nutrition Examination Survey; SE = standard error; w = week. ^a^ Respiratory symptom index developed based on responses to questions from the International Study of Asthma and Allergy in Children. ^b^ Prevalences weighted using PATH Study Wave 2 single-wave weights and based on all Wave 2 adults 18+. The exclusions used throughout the rest of the analyses are not used, so that the PATH Study data are comparable to the NHANES data. ^c^ Prevalences weighted using NHANES 2011–2012 full sample 2-year interview weights. ^d^ None for these two questions include “No” response to question R02_AX0046 (ever wheezing) and/or R02_AX0047 (past 12-month wheezing). ^e^ None/no for these three questions include the “No” option from RDQ070 (past 12-month wheezing).

**Table 2:** Association between four respiratory symptom score cut-off levels and self-reported asthma diagnosis, Wave 2 (2014–2015) PATH Study.

		Predictive Validity								
		Lifetime Asthma *n* = 1829/16,295 AROC = 0.7517			Past-Year Asthma, No Meds *n* = 436/14,902 AROC = 0.8033			Past-Year Asthma, On Meds *n* = 371/14,837 AROC = 0.9057		
Respiratory Symptom Index Score Cut-Off Level	Weighted Prevalence (SE)	Unweighted Sensitivity	Unweighted False Positive Rate	Weighted Unadjusted Odds Ratio (95% CI)	Unweighted Sensitivity	Unweighted False Positive Rate	Weighted Unadjusted Odds Ratio (95% CI)	Unweighted Sensitivity	Unweighted False Positive Rate	Weighted Unadjusted Odds Ratio (95% CI)
≥1	22.8% (0.5)	68.3%	22.3%	10.66 (9.28, 12.24)	77.1%	22.3%	14.19 (9.53, 21.13)	91.9%	22.3%	57.14 (35.55, 91.85)
≥2	10.7% (0.3)	47.1%	10.2%	12.08 (10.59, 13.78)	58.7%	10.2%	18.89 (13.86, 25.74)	80.1%	10.2%	53.75 (38.50, 75.04)
≥3	7.2% (0.3)	35.2%	6.5%	12.37 (10.55, 14.49)	44.7%	6.5%	17.85 (13.13, 24.28)	67.7%	6.5%	43.62 (31.24, 60.90)
≥4	4.6% (0.2)	23.8%	4.0%	11.27 (9.40, 13.52)	26.6%	4.0%	10.87 (7.66, 15.44)	54.2%	4.0%	38.66 (27.86, 53.63)

Abbreviations: PATH = Population Assessment of Tobacco and Health; AROC = area under the receiver operator characteristic (ROC) curve; SE = standard error; CI = confidence interval.

**Table 3:** Questions related to health and functional status for Wave 2 (2014–2015) of the PATH ^a^ Study.

Question	Response Options	PATH Study Variable	Weighted Prevalence (Standard Error)	
**Does your health limit you in any of the following activities?**	**Choose all that apply.**			
Walking up 10 steps	Yes, No	R02_ AX0097_02	Yes	4.2% (0.3)
Walking 3 blocks	Yes, No	R02_ AX0097_03	Yes	5.8% (0.4)
Walking a mile	Yes, No	R02_ AX0097_04	Yes	9.4% (0.4)
In the past 7 days, how would you rate your fatigue on average? By fatigue, we mean feeling unrested or overly tired during the day, no matter how many hours of sleep you’ve had.	1 = None	R02_ AX0101	None	23.8%(0.6)
	2 = Mild		Mild	47.3% (0.6)
	3 = Moderate		Moderate	24.1% (0.5)
	4 = Severe		Severe	4.0% (0.2)
	5 = Very severe		Very Severe	0.8 (0.1)
In general, how would you rate your physical health?	1 = Excellent	R02_ AX0090	Excellent	17.4% (0.5)
	2 = Very good		Very good	39.7% (0.5)
	3= Good		Good	32.5% (0.5)
	4 = Fair		Fair	9.3% (0.3)
	5= Poor		Poor	1.1% (0.1)

^a^ Population Assessment of Tobacco and Health. Unweighted *n* = 16,263 for 10 steps; Unweighted *n* = 16,263 for three blocks; Unweighted *n* = 16,263 for 1 mile; Unweighted *n* = 16,284 for fatigue; Unweighted *n* = 16,291 for physical health.

**Table 4:** Weighted association between functionally important respiratory symptoms and functional outcomes, Wave 2 (2014–2015) of the PATH Study. ^a^

	Respiratory Symptom Index Cut-Off Value				
Functional Outcome	Continuous	≥1	≥2	≥3	≥4
	Adjusted ^b^ Odds Ratio (95% CI)				
**Physical limitations ^c^**					
Walking up 10 steps	1.19 (1.11, 1.28)	1.46 (1.12, 1.89)	1.96 (1.42, 2.68)	2.00 (1.45, 2.76)	2.45 (1.56, 3.22)
Walking 3 blocks	1.25 (1.18, 1.33)	1.77 (1.41, 2.22)	2.10 (1.55, 2.83)	2.19 (1.60, 3.01)	2.89 (2.13, 3.93)
Walking a mile	1.31 (1.25, 1.38)	2.00 (1.65, 2.43)	2.84 (2.31, 3.49)	2.71 (2.14, 3.43)	3.13 (2.39, 4.10)
	Adjusted ^b^ Relative Risk Ratio (95% CI)				
**Fatigue ^d^**					
None	Ref	Ref	Ref	Ref	Ref
Mild	1.24 (1.16, 1.33)	1.76 (1.51, 2.07)	1.85 (1.51, 2.29)	1.83 (1.41, 2.37)	1.99 (1.41, 2.82)
Moderate	1.45 (1.34, 1.56)	2.79 (2.35, 3.31)	3.04 (2.40, 3.86)	2.85 (2.18, 3.72)	3.76 (2.68, 5.27)
Severe	1.67 (1.54, 1.81)	4.19 (3.37, 5.21)	5.35 (4.02, 7.12)	5.25 (3.78, 7.29)	7.04 (4.66, 10.62)
Very severe	1.74 (1.54, 1.96)	3.82 (2.37, 6.17)	6.31 (3.94, 10.11)	7.69 (4.73, 12.49)	10.00 (5.52, 18.11)
	Adjusted ^b^ Relative Risk Ratio (95% CI)				
**Physical health ^e^**					
Excellent	Ref	Ref	Ref	Ref	Ref
Very good	1.19 (1.11, 1.28)	1.48 (1.24, 1.76)	1.78 (1.41, 2.26)	1.48 (1.12, 1.94)	1.58 (1.05, 2.36)
Good	1.46 (1.35, 1.58)	2.53 (2.09, 3.05)	3.55 (2.71, 4.64)	3.14 (2.34, 4.20)	3.64 (2.52, 5.26)
Fair	1.69 (1.54, 1.84)	3.53 (2.82, 4.42)	5.87 (4.39, 7.85)	5.55 (4.07, 7.57)	6.90 (4.42, 10.79)
Poor	1.91 (1.73, 2.11)	6.70 (4.46, 10.07)	8.64 (5.78, 12.93)	9.02 (5.88, 13.83)	11.66 (7.21, 18.83)

Abbreviations: PATH = Population Assessment of Tobacco and Health; CI = confidence interval; Ref = reference group. ^a^ Unweighted *n* = 16,263 for 10 steps; Unweighted *n* = 16,263 for three blocks; Unweighted *n* = 16,263 for 1 mile; Unweighted *n* = 16,284 for fatigue; Unweighted *n* = 16,291 for physical health. ^b^ All models control for age, gender, body mass index, and self-report for any of the following medical conditions: diabetes, cancer, congestive heart failure, and heart attack. Physical limitations models present odds ratios, and fatigue and physical health models present relative risk ratios. ^c^ Does your health limit you in doing any of the following activities? Choose all that apply: Walking up 10 steps, walking 3 blocks, walking a mile. (no, yes). Weighted logistic regression was used to model each outcome (yes vs. no) separately. ^d^ In the past 7 days, how would you rate your fatigue on average? By fatigue, we mean feeling unrested or overly tired during the day, no matter how many hours of sleep you’ve had. (none, mild, moderate, severe, very severe). Weighted multinomial regression was used to model the severity of fatigue as one outcome. ^e^ In general, how would you rate your physical health? (excellent, very good, good, fair, poor). Weighted multinomial regression was used to model the quality of physical health as one outcome.

**Table 5:** Weighted relationship between different thresholds for functionally important respiratory symptoms and association with cigarette exposures, Wave 2 (2014–2015) of the PATH Study. ^a^

	Respiratory Symptom Index Cut-Off Value				
Variable	Continuous	≥1	≥2	≥3	≥4
	Relative risk (95% CI) ^b^				
Current cigarette smoking ^c^					
Never	Ref	Ref	Ref	Ref	Ref
Former	1.34 (1.16, 1.55)	1.28 (1.13, 1.45)	1.37 (1.14, 1.65)	1.32 (1.04, 1.69)	1.49 (1.09, 2.06)
Current nondaily	1.96 (1.67, 2.29)	1.89 (1.65, 2.15)	2.08 (1.70, 2.55)	2.02 (1.58, 2.59)	2.12 (1.48, 3.03)
Current Daily	3.11 (2.75, 3.52)	2.29 (2.08, 2.53)	3.73 (3.22, 4.31)	4.02 (3.36, 4.81)	4.31 (3.36, 5.52)
Pack-years of cigarette smoking (per each additional 5 years)	1.05 (1.03, 1.07)	1.05 (1.03, 1.07)	1.05 (1.03, 1.07)	1.05 (1.02, 1.08)	1.04 (1.01, 1.08)
Second-hand smoke exposure (per each additional 5 h)	1.03 (1.02, 1.04)	1.02 (1.01, 1.03)	1.03 (1.02, 1.04)	1.03 (1.02, 1.04)	1.04 (1.03, 1.06)

Abbreviations: PATH = Population Assessment of Tobacco and Health CI = confidence interval; Ref = reference group. ^a^ Unweighted *n*= 16,295 adult respondents without chronic obstructive pulmonary disease or other non-asthma respiratory disease and with PATH Study longitudinal (all-waves) weights and complete data on all variables listed in Figure 1 Footnote e. ^b^ Adjusted for the other variables in the table. ^c^ Never tobacco user category includes non-established (e.g., lifetime use of <100 cigarettes) users; former established user category user includes all established users (e.g., lifetime use of 100 or more cigarettes) who did not smoke a cigarette in the past 30 days; both current categories include established users (e.g., lifetime use of 100 or more cigarettes) who did smoke a cigarette in the past 30 days.

## Data Availability

Data from the PATH Study Wave 1 to Wave 3 are available for download as Restricted Use Files (https://www.icpsr.umich.edu/icpsrweb/NAHDAP/studies/36231). Request guidelines and conditions of use are available at the website above.
